# The Blood Levels of Trace Elements Are Lower in Children With Tic Disorder: Results From a Retrospective Study

**DOI:** 10.3389/fneur.2019.01324

**Published:** 2019-12-17

**Authors:** Ruiying Qian, Ying Ma, Liuqing You, Yanmin Zhao, Shuxian Li, Jue Shen, Lihua Jiang, Cuiwei Yang, Peifang Jiang, Zhefeng Yuan, Feng Gao, Shanshan Mao

**Affiliations:** ^1^Department of Neurology, The Children's Hospital, Zhejiang University School of Medicine, National Clinical Research Center for Child Health, Hangzhou, China; ^2^Department of Epidemiology and Biostatistics, Zhejiang University School of Public Health, Hangzhou, China; ^3^Department of General Practice, Community Health Service Center, Hangzhou, China; ^4^Department of Pulmonology, The Children's Hospital, Zhejiang University School of Medicine, National Clinical Research Center for Child Health, Hangzhou, China

**Keywords:** tic disorders (TD), trace elements, zinc (Zn), copper (Cu), iron (Fe)

## Abstract

**Background:** Tic disorders (TD) are common neuropsychiatric disorders among children and adolescents. It is controversial that trace elements may participate in the pathogenesis of TD. Our study aimed to investigate the trace elements status of zinc (Zn), copper (Cu), iron (Fe), and magnesium (Mg) in children with TD, in comparison to healthy controls.

**Methods:** The medical records of eligible TD children and normal healthy children from January 1 to December 31, 2018 in the outpatient clinic were retrospectively reviewed. The clinical information of all subjects were collected including age, gender, diagnosis, previous health records, and serum trace elements level (Cu, Zn, Fe, Mg) at the time of diagnosis before initiating treatment.

**Results:** In total, 1204 TD children (7.63 ± 2.45 years) and 1,220 healthy children (7.27 ± 3.15 years) who were divided into two gender and three age groups (2–4years, 5–9years, ≥10 years) were reviewed in our study. Our study showed that TD children generally had lower whole blood levels of Zn, Cu, Fe than the normal controls (*P* < 0.01). No significant difference was observed in whole blood levels of Mg. After adjusting for gender, the trends still remained. Further analysis was performed according to age, the trends still remained in Zn and Fe in all age groups (*P* < 0.05). However, we observed an almost significantly (*P* = 0.055) lower level of Cu in TD of 2–4 years group while significant differences in other two groups (*P* < 0.01). Further multiple linear regression and point biserial correlation showed that the lower blood levels of Zn, Cu, and Fe were correlated with the incidence of TD.

**Conclusion:** The present results indicated that lower blood levels of zinc, iron, copper were associated with TD. Trace elements may be used as an auxiliary treatment for TD and need to be further explored.

## Introduction

Tic disorders (TD), including transient tic disorder (TTD), chronic motor or vocal tic disorder (CTD), and Tourette syndrome (TS), are common neuropsychiatric disorders that often occur in children and adolescents. They are characterized by sudden, rapid, unconscious motor movements or uttered sounds that involve discrete muscle groups. Transient tics occur in up to 25% of healthy children. The prevalence of chronic tic disorder is about 1–3% and that for TS varies from 0.1 to 1%, with a male: female ratio of approximately 4:1 (1, 2). The age of tic onset is mainly in early kindergarten and school years with a range from 3 to 11 years and its severity usually peaks at 12 years old. Most patients show an ability to suppress tics for short periods but report an increase of symptoms shortly after. About 5–10% patients exhibit a persistent impairing system or functioning that lasts into adulthood, resulting in a permanent impairment of social and academic function which could affect one's lifetime achievement (3).

TD is currently recognized as a polygenic genetic disorder and is affected by a variety of environmental factors (4, 5). An inherited nature for TD supported by findings, including a population-based heritability estimate of 0.77 (a value of 1 indicating 100% heritability), a 15-fold increased risk of developing Tourette syndrome/chronic motor or vocal tic disorder in siblings of individuals with Tourette syndrome, a positive family history for tics in about one-half of patients, and monozygotic twins having an 86% concordance rate with chronic motor or vocal tic disorder compared to 20% in dizygotic twins (6). Although hundreds of genes appear to be involved, only a small proportion of risk has been explained. A huge study of shared data from over a million people with various neurologic and psychiatric disorders revealed that TS shares common variant genetic risk with obsessive compulsive disorder (OCD), major depression, and migraine, especially migraine with aura (7, 8). There is much literature exploring immune dysregulation in TS, including microglial dysfunction, reduced numbers of regulatory T cells, altered gamma globulin, and an increased response to pathogens, and these have been proposed as potential contributing etiologic factors for tics (9). In some cases, behavioral changes and tics occur after streptococcal infection and are currently thought to be associated with autoimmune responses following streptococcal infection. There is some evidence that these patients can respond to antibiotics or immune therapy, which is still controversial (10). Other researches on pathogenesis involve prenatal/perinatal infections, psychosocial situation and environmental factors (9). However, the mechanism of TD pathogenies has not been found yet. Therefore, the treatment of TD could be more complex and diversified as well.

In current clinics, treatment of tic disorder mainly depends on medicines (11). The conventional medicines increase the activity of dopamine and norepinephrine and those stimulants have a high effectiveness in TD treatment. However, it also has noticeable side effects, such as: loss of appetite and weight, growth inhibition, abdominal pain, headaches, sleeping problems, increased blood pressure, and even addiction. Therefore, scientists and parents keep seeking for alternative tools of controlling TD. Trace elements such as copper (Cu), zinc (Zn), iron (Fe), and magnesium (Mg) which are currently known to be essential for brain development and physiology, including neurotransmitter synthesis (12), concentrations in the basal ganglia (13), neurotransmitters and dopamine metabolism (14, 15). They are proposed to be one of possible key factors of TD pathology (7), and received more and considerable attention from the scientific community. TD coexists with many common comorbidities, the most common of which is attention deficit hyperactivity disorder (ADHD). Some studies have found that serum Zn and Fe levels are lower in children with ADHD than those in normal children, but this phenomenon has not been observed in Mg or Cu (16). Moreover, the results from existing literatures are not always consistent and the parameters of the state of trace elements are also controversial (17). Our study analyzed the relationship between trace elements and TD in children based on a large sample size. We expect that the study may inspire the development of new approaches to treat TD.

## Methods

### Study Design

In this study we measured the levels of trace elements in children with TD and healthy children aged 2–15 years in order to assess whether the levels of trace elements in children with TD are different.

### Study Procedure

Our study retrospectively reviewed the electronic medical record database of pediatric patients newly diagnosed with TD through comprehensive evaluation and control subjects at the Children's Hospital of Zhejiang University School of Medicine between January 1, 2018 and December 31, 2018.

The children included in the TD group should meet the following criteria: (1) diagnosed as TD through a comprehensive assessment according to the Diagnostic and Statistical Manual of Mental Disorders, Fifth Edition (DSM-5): one or more motor tics or vocal tics occurring several times daily; (2) between the ages of 2 and 15 years old; (3) without history of TD treatment; (4) excluding similar symptoms caused by other physical diseases and substance use. Individuals were excluded if they had (1) a neuroimaging diagnosis with brain structural abnormalities; (2) history of taking dopamine receptor blockers, cocaine and other drugs for controlling TD; (3) combined with other diseases such as cardiovascular diseases, severe malnutrition, severe digestive system diseases such as severe gastrointestinal stenosis and dysphagia. The control group was recruited from children who underwent health checkups within the same time period. TD and other serious medical diseases were excluded by detailed examination.

Clinical information of all subjects were collected including age, gender, diagnosis, family history of TD, previous health record, and serum trace element level at the time of diagnosis before initiating treatment.

A total of 1,204 children were enrolled in TD group and 1,220 children were enrolled in control group. The characteristics of the subjects are shown in Table 1. The mean age of the controls and children with TD was 7.27 ± 3.15 and 7.63 ± 2.45 years old, respectively. For further analysis, the children were divided either based on gender into two groups (1,669 males and 755 females) or based on age into three groups (2–4 years, *n* = 490; 5–9 years, *n* = 1,457; ≥10 years, *n* = 477).

**Table 1:** Basic characteristics of children in the TD group and normal control group.

	**Control group *N* (%)**	**TD group** ***N* (%)**	***P*-value**
Age, years		
Mean ± *SD*	7.27 ± 3.15	7.63 ± 2.45	0.001
2~	325 (26.64%)	165 (13.70%)	<0.001
5~	636 (52.13%)	821 (68.19%)	
10~	259 (21.23%)	218 (18.11%)	
Sex		
Male	732 (60.00%)	937 (77.82%)	<0.001
Female	488 (40.00%)	267 (22.18%)	

### Trace Element Detection

Trace metal analysis were performed with a multichannel atomic absorption spectrophotometer-MB5 (AAS; Beijing Purkinje General Instrument Co., Ltd., Beijing, China). The detection thresholds of these instruments for Zn, Cu, Fe, and Mg are 0.01 μmol/l, 0.01 μmol/l, 0.01 mmol/l, and 0.01 mmol/l, respectively.

### Data Analysis

Statistical analysis was performed using SPSS (version 20.0) statistical software. The data were expressed as mean ± standard deviation. The comparison between two groups was performed by independent sample *t*-test. The comparison between multiple groups was analyzed by one-way ANOVA. The comparison between groups was performed by SHK-q test. The counting data were presented as percentage (%). The chi-square test was used to compare the demographic data of two groups. Multiple linear regression and point biserial correlation was used to analyze the relationship between TD status and serum trace elements. *P* < 0.05 was considered statistically significant.

### Ethics Statement

The research protocol was approved by the institutional review committee of the Children's Hospital of Zhejiang University School of Medicine (2019-IRB-090). According to nature of the study, informed consent was waived by the Institutional Review Board.

## Results

Two thousand four hundred twenty-four children were included into this study. Of these, 1,669 (68.85%) children were males and the mean ± SD age was (7.54 ± 2.81) years. The basic characteristics of the samples are shown in Table 1. TD group was older than control group (7.63 vs. 7.27, *P* < 0.001) and had a higher proportion of males (77.82 vs. 60.00%, *P* < 0.001).

Differences between children in TD group and control group in terms of trace element level were presented in Table 2. TD group had a lower levels of Zn, Cu and Fe than the control group (78.90 vs. 83.90 μmol/l, 17.80 vs. 18.50 μmol/l, and 8.47 vs. 8.80 mmol/l, all *P* < 0.001). No statistical difference was observed regarding the level of Mg. After adjusting for gender and age, the trends still remained except “2–4” age group. We observed a trend of lower level of Cu in TD of 2–4 years group that is close to significance (*P* = 0.055), while it was significantly lower in the other two aging groups.

**Table 2:** Trace element level of children in TD and control group.

	**Control group (Mean ± *SD*)**	**TD group** **(Mean ± *SD*)**	***P*-value**
**TOTAL**			
Zn (μmol/l)	83.90 ± 12.10	78.90 ± 11.50	<0.001
Cu (μmol/l)	18.50 ± 3.54	17.80 ± 3.28	<0.001
Fe (mmol/l)	8.80 ± 0.94	8.47 ± 0.86	<0.001
Mg (mmol/l)	1.57 ± 0.17	1.58 ± 0.19	0.318
**SEX**			
**Male**			
Zn (μmol/l)	84.30 ± 12.20	78.70 ± 11.40	<0.001
Cu (μmol/l)	18.60 ± 3.54	17.80 ± 3.27	<0.001
Fe (mmol/l)	8.85 ± 0.967	8.49 ± 0.83	<0.001
Mg (mmol/l)	1.57 ± 0.175	1.58 ± 0.19	0.357
**Female**			
Zn (μmol/l)	83.30 ± 11.90	80.00 ± 11.70	<0.001
Cu (μmol/l)	18.40 ± 3.54	17.70 ± 3.31	0.005
Fe (mmol/l)	8.73 ± 0.89	8.40 ± 0.95	<0.001
Mg (mmol/l)	1.57 ± 0.17	1.57 ± 0.19	0.949
**AGE**			
**2~**			
Zn (μmol/l)	77.10 ± 10.3	74.10 ± 11.30	0.005
Cu (μmol/l)	19.20 ± 3.57	18.60 ± 3.37	0.055
Fe (mmol/l)	8.42 ± 0.77	8.27 ± 0.77	0.039
Mg (mmol/l)	1.56 ± 0.16	1.60 ± 0.19	0.028
**5~**			
Zn (μmol/l)	84.70 ± 11.4	79.20 ± 11.00	<0.001
Cu (μmol/l)	18.40 ± 3.38	17.80 ± 3.28	<0.001
Fe (mmol/l)	8.84 ± 0.94	8.45 ± 0.93	<0.001
Mg (mmol/l)	1.57 ± 0.18	1.57 ± 0.18	0.793
**10~**			
Zn (μmol/l)	90.50 ± 11.70	81.7 ± 12.3	<0.001
Cu (μmol/l)	17.80 ± 3.70	17.1 ± 3.06	<0.001
Fe (mmol/l)	9.18 ± 0.97	8.71 ± 0.95	0.032
Mg (mmol/l)	1.58 ± 0.18	1.60 ± 0.19	0.306

Point-biserial correlation of TD and the blood level of trace elements was shown in Table 3. The blood levels of Zn, Cu, and Fe were negatively associated with TD (*r* = −0.205, −0.181, and −0.106, all *P* < 0.001).

**Table 3:** Point-biserial correlation of TD and the blood level of trace elements.

	***r***	***P*-value**
Zn	−0.205	<0.001
Fe	−0.181	<0.001
Cu	−0.106	<0.001
Mg	0.02	0.3175

All element quantities were further transformed into categorical variables according to the clinical reference categories. The distributions of categorical trace element levels in two groups are given in Table 4. There existed significantly difference in categorical zinc distribution between two groups (*P* < 0.001). And the proportion of zinc deficiency in TD group was higher than that in control group (40.28 vs. 22.05%). No other statistical differences were observed in other categorical elements. The odds ratios (ORs) and 95% confidence intervals (95%CIs) for the relationship between zinc deficiency and the risk of TD are presented in Table 5. Zinc deficiency was independently and significantly associated with an increased risk of TD (OR = 2.52, 95%CI 2.09–3.03), with adjustment for age and sex.

**Table 4:** The relationship between the level of trace elements and TD.

**Elements**	**Control group *N* (%)**	**TD group** ***N* (%)**	***P*-value**
Zn			<0.001
Low	269 (22.05%)	485 (40.28%)	
Normal	951 (77.95%)	719 (59.72%)	
Cu			0.4967
Low	0 (0)	1 (0.08%)	
Normal	1220 (100%)	1203 (99.92%)	
Fe			0.5646
Low	4 (0.33%)	3 (0.25%)	
Normal	1216 (99.67%)	1200 (99.67%)	
High	0 (0)	1 (0.08%)	
Mg			0.2466
Normal	1220 (100%)	1202 (99.83%)	
High	0 (0)	2 (0.17%)	

**Table 5:** Logistic regression analysis of zinc deficiency with TD.

	**Crude OR (95%CI)**	**Adjusted OR (95%CI)^a^**
**Zn**		
Low	2.38 (2.00–2.85)	2.52 (2.09–3.03)
Normal	1.00 (ref)	1.00 (ref)

a *Adjusted for age and gender*.

## Discussion

Tic disorder is a neurodevelopmental disorder in children characterized by motor and vocal tics. The age of onset is mainly in early kindergarten and school years, ranging from 3 to 11 years (mean age is between 6 and 7 years). Tics can only be expressed as simple actions such as blinking, nose twitching, or complex movements such as jumping, uttering words, etc. Its severity usually peaks at around 12 years, with reducing frequency and severity at the end of adolescence (18). Epidemiological studies have shown that males account for the majority of TD patients (ratio 4:1) (19). About 5–10% patients will exhibit persistent tics, which in turn leads to severe difficulties in social functioning (20). Emotional states such as excitement, depression, stress and anxiety are convinced to increase the severity of tics.

TD is currently recognized as a polygenic genetic disorder and is affected by a variety of environmental factors such as prenatal and perinatal noxious events, infections, and so on (18, 21). In the past few years, TD's genetic research has made great progress, however, more and more studies have shown that environmental factors contribute not only to the gene–environment interaction but also at least to the disorders' severity and course.

Antipsychotics are the oldest and most effective treatment for tic disorder. Although most randomized controlled trials of antipsychotic treatment of tic disorder have shown superior efficacy over placebo, there are also many potentially serious adverse effects, including drug-induced dyskinesia, metabolism, and hormonal effects (22). In the past 15 years, patients with TD who have failed to respond to drugs and behavioral interventions can consider to try neuromodulation strategies primarily on, the deep brain stimulation (DBS) (11, 23). The evidence of efficacy and safety of DBS in TS is based fundamentally on two larger studies targeting the globus pallidus pars interna (Gpi) and other four studies that used Gpi and thalamic intralaminar nuclei as targets and had smaller sample sizes (24–28). However, due to recent differences in the results of randomized controlled trials for Gpi, there is still uncertainty in target selection. Another obstacle associated with DBS in TS is the relatively high frequency of post-operative infection complications, which may be associated with severe head twitching, self-manipulation of surgical wounds, or overreaction of immune inflammatory responses. In the future, there is an urgent need to develop more effective and safety treatments to control tics.

The patients' characteristics of children with TD in our cohort were consistent with those described in the literature (1). The mean age at diagnosis in our center was 7.63 years, with the highest proportion of children aged 5–9 years, which is consistent with previous epidemiological studies (1). Male preponderance is also consistent with the general childhood population of TD (29).

Up to date, very few studies were devoted to exploring whether the level of trace elements changed in children with TD, there is still no consensus on this conclusion (30). Avrahami et al. (17) reported that serum ferritin levels are lower in children with TD compared with those without tics (TD). Ghosh and Burkman noted that the children with TD who had low serum ferritin levels (≤50 ng/mL) tended to have higher tic severity scores, but they did not obtain significant statistical differences (31), neither of above two studies involved other trace elements. Both two studies have small sample sizes and lack the normal healthy control groups. None of above found a relationship between serum iron (another important and sensitive parameter to estimate total body iron store) and tic disorder.TD coexists with many common comorbidities, including obsessive compulsive disorder (OCD) (26%), attention deficit hyperactive disorder (ADHD) (63%), anxiety (49%), depression (25%) (32), and sleep problems (60%) (33). Some studies had found that the level of trace elements in serum of children with ADHD is lower than that of control groups (29). Yang et al. (16) studied the most common four elements (Cu, Zn, Fe, Mg) routinely measured in clinical practice and reported that lower zinc levels were found in children with ADHD when compared with the normal control group while the other three elements didn't find such differences. Sun et al. (34) followed a systematic research for case-control studies on the serum zinc levels in Chinese children with ADHD published between 2000 and 2015 and obtained the similar conclusion as above.

In our study, the first advantage is that the designed subjects had a wide range of ages, we controlled inclusion and exclusion criteria to minimize the effects of other diseases, especially digestive diseases, on the intake of trace elements; second, we also measured four trace elements that are often collected in clinical practice; the third advantage is the large sample size, which can minimize the selective bias; fourthly, due to differences in age and gender distribution between the control and the TD group. In order to make the results credible, we've analyzed the two groups according to age and gender, respectively. These advantages make our results more powerful. In our study, we found that the average zinc level in the TD group was lower than that in the control group. The difference remained when adjusting the factor of gender and age, respectively. This conclusion is the same as previous researches (16). In addition, further point biserial correlation analysis of serum zinc and TD showed that the *r* value was negative, suggesting a negative correlation between serum zinc and TD. Interestingly, when we divided serum zinc into normal and deficiency according to the corresponding reference range, we found that a significant proportion of children with TD had zinc deficiency. Further correlation analysis showed that zinc deficiency was positively correlated with TD. The role of zinc in the pathophysiological process of TD is still unclear. Animal experiments and human studies suggest that there are several possible pathways that could be involved (35, 36): (1) zinc is an essential cofactor for over 300 enzymes (metalloenzymes and metalloenzyme complexes) required for the metabolism of carbohydrates, fatty acids, proteins, and nucleic acids. The metabolism of these nutrients is crucial for brain structure development and functional maintenance (37); (2) zinc is also essential for the production and regulation of the pineal hormone melatonin which helps to regulate dopamine function and is widely recognized as a key factor in the treatment of TD (38, 39); (3) zinc can also bind to and regulate dopamine transporters, which are targets of psychostimulants for the treatment of ADHD (40). Based on these evidence, we believe that zinc supplementation may be an auxiliary treatment for TD children who with zinc deficiency. Some studies suggest that zinc as part of a mineral supplement may be a safe and effective intervention for ADHD (41). A large sample prospective cohort study is needed to confirm the efficacy and identify the real and efficient dose of zinc in TD.

Iron is another important trace element in the body and plays an important role in the development of the brain. Previous studies have found that iron deficiency affects children's cognitive, motor, and social functions. Changes in iron levels are associated with dopamine-related dyskinesias, such as restless legs syndrome (42). We selected serum iron which is relatively easy to obtain as a research object. Our study shows that serum iron levels in children with TD are lower than controls. After adjusting the factor of gender and age, respectively, the conclusion which is different from previous research results, is still remained (33). Further point biserial correlation analysis showed a negative correlation between serum iron and TD. The specific mechanism of TD and iron is not completely clear. Previous studies have found that iron is a major cofactor of the tyrosine hydroxylase enzyme which is a rate-limiting step in dopamine synthesis (43). Second, iron deficiency is associated with decreased density and activity of dopamine transporters, which in turn leads to an increase in extracellular dopamine and a decrease in dopamine receptors in the striatum (44, 45). Third, iron deficiency may lead to basal ganglia dysfunction (46). Fourth, the imbalance of inhibitory/excitatory neurotransmitters is considered to be one of the main factors in the development of TD (47). Gamma-aminobutyric acid (GABA) is a major inhibitory neurotransmitter in the mammalian central nervous system and affects iron levels in the brain (48). Patients with TD have lower levels of GABA, which may result in a decrease in iron concentration in the basal ganglia (48). Fifth, imaging studies have shown that the level of thalamic iron in children with TD is reduced, which may be a potential cause of TD (49).

It is worth noting that only a small number of patients in our study have serum iron deficiency, while serum iron in most children with TD is within the normal reference range. Serum iron levels may not be consistent with iron levels in the brain. Therefore, serum iron may not be the optimal indicator for determining iron storage in children with TD in clinical work and need to be determined. Ghosh and Burkman noted that the iron sufficient patients receiving iron supplementations also showed improvement in their tic severity with time, while the tics in those without supplementation remained unchanged (33). It is suggested that iron sufficient patients may have relative iron deficiency in the brain. Therefore, for patients with normal but low serum iron levels, that whether regular iron supplementation is needed deserves to be further exploration.

Copper is essential for many enzymes, which play important roles in neurophysiology, including cellular antioxidants, dopamine metabolism, norepinephrine, catecholamine degradation (50). Excess copper levels and copper-mediated neurotoxicity may be associated with the formation of a copper-dopamine complex which oxidizes dopamine and might be related to depression and other mental problems such as schizophrenia, learning disabilities, hyperactivity, and so on (51, 52). At present, there are few studies to pay attention to the relationship between copper and tic disorder. Our study showed that serum copper levels in the TD group were lower than that in the control group which is different from previous studies (16). Further analysis based on age, the other two groups obtained similar results as above, whereas for the 2–4 age group, there was no difference in copper levels between the two groups. It is worth noting that there are differences in the values of copper levels between the TD and the control group, but not statistically different, which may be related to the small sample size of this group. Further point biserial correlation analysis showed a negative correlation between serum copper and TD. The relationship between copper and TD deserves further discussion.

Our study has several limitations: (1) all the children in this study are from a single center, and we selected serum trace element levels as the research object, which may not reflect the level of trace elements in the actual brain. (2) This study only reveal the relationship of trace element with tic disorder, it is still unclear whether supplementation of corresponding trace elements may help to control and improve symptoms of tics, further researches are needed. (3) TD always coexists with many common comorbidities. Our study did not further classify the subjects according to the comorbidities. The relationship between different comorbidities and trace elements still needs to be further explored. These limitations should be taken into consideration when the results interpreted.

## Conclusion

Our study shows that zinc deficiency was related to occurrence of TD and blood copper/iron levels in patients with TD were lower than those in the normal control group. Therefore, whether zinc supplementation can be used as an effective auxiliary means for controlling tics and whether children with TD need regular supplementation of trace element (Cu/Fe), deserve further exploration. We suggest a double-blind, placebo-controlled prospective study to reach a definite conclusion.

## Data Availability Statement

The raw data supporting the conclusions of this article will be made available by the authors, without undue reservation, to any qualified researcher.

## Ethics Statement

The studies involving human participants were reviewed and approved by institutional review committee of the Children's Hospital of Zhejiang University School of Medicine (2019-IRB-090). Written informed consent from the participants' legal guardian/next of kin was not required to participate in this study in accordance with the national legislation and the institutional requirements.

## Author Contributions

RQ, YM, LY, SM, and FG participated in the design of the study, collected and analyzed the data, and drafted the manuscript. RQ, LY, SM, YZ, SL, JS, and ZY collected the data. LY, LJ, CY, and PJ was responsible for analysis, analyzed the data, and contributed in drafting the manuscript. RQ, YM, and SM contributed in drafting the manuscript. All authors read and approved the final manuscript.

### Conflict of Interest

The authors declare that the research was conducted in the absence of any commercial or financial relationships that could be construed as a potential conflict of interest.
